# The Influence of demographic variables on the prevalence and severity of tooth wear in a Trinidadian population

**DOI:** 10.3389/froh.2025.1516137

**Published:** 2025-07-07

**Authors:** Shivaughn Maria Marchan, Reisha N. Rafeek, William A. J. Smith

**Affiliations:** Unit of Restorative Dentistry School of Dentistry, The University of the West Indies St. Augustine, St. Augustine, Trinidad and Tobago

**Keywords:** demographic variables, tooth wear, ethnicity, sex, age

## Abstract

**Introduction:**

This study aimed to assess the prevalence and severity of wear in a Trinidadian adult patient population based on the demographic variables of age, sex and ethnicity.

**Methods:**

Secondary data, stored in an institutional repository as an SPSS file were re-coded. Age was re-coded to stratify the sample into five age groups. A new variable denoting the overall severity of tooth wear was added using the highest score for any examined tooth surface. Data for sex and ethnicity was not re-coded. Likelihood ratios on cross-tabulated data with an alpha level of 0.05 were used to ascertain significant differences in the overall severity of tooth wear for demographic variables. Cross-tabulations were also completed between demographic variables and individual scores for each examined surface in the upper and lower anterior sextants.

**Results:**

There was a highly statistically significant association (*p* < 0.01) between age and the overall severity score of tooth wear in this sample patient population with 64% of 18–30-year-olds having no wear while only 22% of the over 60 age range showed no wear. There were no significant associations on cross-tabulated data for the overall severity score of wear and sex or ethnicity (*p* > 0.05). However, data on tooth wear of specific tooth surfaces when cross-tabulated with sex and gender showed significant associations (*p* < 0.05).

**Conclusion:**

Age appears to influence the prevalence and severity of overall wear. The severity of wear on the incisal edges of specific teeth appears to be influenced, in part, by the demographic variables of sex and ethnicity.

## Introduction

The worldwide prevalence rates for tooth wear vary from 29% to 60% depending on the geographical region of the studied sample population (1). Tooth wear is considered a significant oral condition important to public health practitioners and dental clinicians. Much of the epidemiological research on tooth wear has examined the association between etiological factors such as diet and habits and the prevalence of tooth wear in various populations (2, 3). Epidemiological research has also examined trends in prevalence in specific populations (4, 5). Several studies have shown a disproportionate prevalence of tooth wear in males compared to females (6, 7). The severity of tooth wear tends to worsen in older populations given the cumulative effects of exposure to an acidic diet or conditions causing contact of gastric acid with teeth and normal physiological wear of tooth-to-tooth contact. Comparative assessment of the prevalence and severity of tooth wear based on ethnicity has not been exhaustively explored in peer-reviewed literature.

Rafeek et al. concluded the prevalence rate of tooth wear in a patient population attending a teaching hospital in Trinidad was 72%, with 52% of the sample exhibiting mild wear (8). Gender, age, and ethnicity were collected as part of this study, however, the prevalence and severity of observed wear based on demographic variables of gender and ethnicity were not explored statistically (8). When age was examined as a demographic variable, there was a strong association between tooth wear and age using the inferential statistic of Odds Ratio (8). However, there is anecdotal clinical evidence that the prevalence and severity of tooth wear in this Trinidadian population predominately affects East Indians more than other ethnic groups in the population. Given the demographic profile of the population of Trinidad; in terms of ethnicity where there are approximately equal proportions of three major ethnic groups a comparative evaluation of the prevalence and severity of tooth wear is possible. The three dominant ethnic groups in Trinidad are as follows: Afro-Trinidadians, predominantly from Western Africa, Indo-Trinidadians, predominantly from South-East Asia, and a Mixed population of Afro and Indo-Trinidadians (9).

The present study aimed to assess the prevalence and severity of tooth wear in the Trinidadian adult patient population in different age groups, for both sexes, and the three dominant ethnicities in the Trinidadian population.

## Methods

The original SPSS data from Rafeek et al, stored in an institutional repository, was used to facilitate this data analysis. One hundred fifty-five participants were examined as part of the original research from September 2002 to March 2003. The tooth wear index, developed by Smith and Knight and modified for the 1998 United Kingdom Adult Dental Survey, was used to ascertain the severity of wear on the teeth of the upper and lower anterior sextants as shown in Table 1. The descriptors for the severity of wear (none, mild, moderate, severe) are included in Table 1. The original research project involved three trained and calibrated examiners who ascertained the severity of tooth wear on the prescribed surfaces of teeth in the upper and lower anterior sextant. The buccal, lingual, and incisal edges of maxillary teeth and the incisal edges of mandibular teeth were examined, representing the teeth and surfaces examined in the 1998 UK Adult Dental Survey.

**Table 1:** Scoring criteria used in the assessment of tooth wear.

Score	Criteria
0	Sound; Any TSL is restricted to enamel and does not extend into dentine
1	Loss of Enamel, just exposing dentine: equivalent to mild TSL
2	Loss of enamel >⅓ of surface area: equivalent to moderate TSL Incisal
3	Complete Loss of enamel on a surface with pulp exposure or secondary dentine: equivalent to severe TSL Incisal Pulp exposure/exposure of secondary dentine
8	Fractured Tooth
9	Unscorable: Crown/Bridge/ no visible incisal edge/tip

The SPSS data for age was re-coded to stratify the sample population into one of five age groups (18–30 years, 31–40 years, 41–50 years, 51–60 years, and over 60 years). A new variable denoting the overall severity of tooth wear was added using the highest score for any examined tooth surface to represent each participant's overall severity of tooth wear. Data for sex and ethnicity was not re-coded. Likelihood ratios on cross-tabulated data with an alpha level of 0.05 were used to ascertain significant differences in the overall severity of tooth wear for demographic variables. Cross-tabulations were also completed between demographic variables (age, sex, and gender) and individual scores for each examined surface in the upper and lower anterior sextants.

## Results

### Age

Table 2 shows the prevalence and severity of tooth wear in this sample by demographic group. There was a highly statistically significant difference (*p* < 0.01) between age and the overall severity score of tooth wear in this patient sample population with 64% of 18–30-year-olds having no wear while only 22% of the over 60 age range showed no wear. Considering mild wear, 62.5% of 31–40-year-olds showed mild wear compared to 60% of 41–50-year-olds and 59.0% of 51–60-year-olds. Severe wear was noted in 3% of persons in the 51–60 age range and in 11% of persons over 60 years.

**Table 2:** Prevalence and severity of tooth wear based on demographic variables.

Characteristics	*n*	None	Mild	Moderate	Severe (%)
Age (*n* = 155)					
18–30 years	42	64.0	31.0	5.0	0.0
31–40 years	32	22.0	62.5	12.5	3.0
41–50 years	40	15.0	60.0	18.0	7.0
51–60 years	32	13.0	59.0	25.0	3.0
Over 60 years	9	22.0	33.0	33.0	11.0
Sex (*n* = 148)					
Male	98	28.6	44.9	20.4	6.1
Female	50	30.6	56.1	10.2	3.1
Missing	8				
Race/Ethnicity (*n* = 151)					
Afro-Trinidadian	56	34.0	53.6	10.7	1.7
Indo-Trinidadian	50	20.4	51.0	22.4	6.2
Mixed	45	38.8	44.0	15.0	2.2

### Sex

There were no significant associations on cross-tabulated data for the overall severity score of tooth wear and sex (*p* = 0.27). However, when sex was cross-tabulated with the scores for the individual surfaces of teeth significant associations were noted for the incisal edges of three teeth (Table 3).

**Table 3:** Statistically significant associations between tooth wear and demographic variables.

Demographic variable	Tooth/surface	*p*-value
Sex	Maxillary left lateral (incisal)	0.06
Sex	Mandibular right canine (incisal)	0.03
Sex	Mandibuar left canine (incisal)	0.03
Ethnicity	Maxillary left canine (incisal)	0.048
Ethnicity	Maxillary right canine (incisal)	0.048
Age	Overall severity	<0.001

### Ethnicity

Cross-tabulated data showed no significance for the overall severity score of wear and ethnicity (*p* = 0.63). Significant associations are presented for individual surfaces of teeth cross-tabulated with ethnicity in Table 3. For the upper left canine, persons identifying as predominantly Afro-Trinidadian showed no moderate or severe wear. For those identifying as Indo-Trinidadian, 10.6% demonstrated moderate wear while 6.4% demonstrated severe wear. Of persons identified as mixed, 0% exhibited moderate wear while 2.2% exhibited severe wear.

For the upper right canine, 74.5% of Afro- and 80% of Mixed-Trinidadians showed no evidence of wear while only 48.9% of Indo-Trinidadians showed no evidence of wear. Mild wear was noted in 23.5% of Afro-Trinidadians, 33% of Indo-Trinidadians, and 20% of Mixed-Trinidadians. Moderate wear was noted in 2% of Afro-Trinidadians, 9% of Indo-Trinidadians, and 0% of Mixed-Trinidadians. Severe wear was noted in 2.3% of Indo-Trinidadians.

## Discussion

This work broadens the knowledge base on the relationship between demographic variables and tooth wear. While the original work identified a significant association between age and tooth wear, this new work demonstrated increasing severity with age. These results align with the conclusions of several other researchers (1, 2, 10). Schlueter and Tveit stated that most teeth start showing the pathological effects of wear mechanics in middle age (7). Work completed in the United States with an adult patient sample concluded that prevalence and severity increased with age (2). Researchers in the Netherlands have noted the number of teeth affected by tooth wear increased with increasing age (10). In this same Dutch study, the overall prevalence of severe tooth wear, at 6%, was considered rare; however, in the Trinidadian patient population, the overall prevalence of severe wear in the oldest age group of over 60 years was 11%, almost twice the Dutch rate.

The results suggest there may be a difference in the severity of wear noted between the dominant ethnic groups in Trinidad. Previous research in a cohort of children from the United States demonstrated that African Americans despite higher consumption rates for erosive beverages had the lowest mean rates for dental erosion compared to Hispanics and White Persons (11). Possible explanations could be related to the relative thickness of the enamel of Afro-Trinidadians. Persons of African ancestry in the United States have been shown to have larger teeth and thicker enamel compared to non-black individuals (12, 13). This is due to variations in the expression of the ENAM gene which codes for enamelin, the enamel matrix protein essential to the formation of tooth enamel (14). Thicker enamel would be more resistant to the temporally cumulative effect of attrition, erosion, and abrasion. Afro-Trinidadians, like African-Americans originated in the same geographical regions of Western Africa. In this Trinidadian patient population, persons of mixed ethnicity appeared to have the same protective effect as African ethnicity having higher prevalence rates of no wear and lower rates of mild, moderate, and severe wear compared to Indo-Trinidadians.

When considering sex, there were higher prevalence rates for males with moderate and severe tooth wear compared with mild wear. This was statistically significant for the incisal edges of some teeth. This is comparable to findings in Sub-Saharan populations, where males had higher rates of wear with authors postulating higher bite forces as the possible etiology (15). Notably, work on Indian populations also shows an increased prevalence of tooth wear in male subjects (16). Females, however in the African diaspora have been shown to have thicker enamel than male counterparts (14). This may have accounted for the favorable finding of higher prevalence rates of no/mild wear across the female sample.

Limitations to this work include the age of the data. New data collection with a larger cohort is planned to assess whether prevalence rates and severity have changed or remained stable in the Trinidadian population. The same Tooth Wear Index will be used to collect data for comparative purposes. The size of the original data set proved insufficient to assess the interaction between the three demographic variables on the severity using statistical tests such as multiple regression analysis. Data from a larger patient cohort will have to be collected to assist with such statistical analysis.

## Conclusions

Within the limitations of this research, age appears to influence the prevalence and severity of overall wear. The severity of wear on the incisal edges of specific teeth appears to be influenced, in part, by the demographic variables of sex and ethnicity.

## Data Availability

The data analyzed in this study is subject to the following licenses/restrictions: None. Requests to access these datasets should be directed to shivaughn.marchan@sta.uwi.edu.
